# Characterization and correction of center‐frequency effects in X‐nuclear eddy current compensations on a clinical MR system

**DOI:** 10.1002/mrm.28607

**Published:** 2020-12-04

**Authors:** Mary A. McLean, R. Scott Hinks, Joshua D. Kaggie, Ramona Woitek, Frank Riemer, Martin J. Graves, Dominick J. O. McIntyre, Ferdia A. Gallagher, Rolf F. Schulte

**Affiliations:** ^1^ Department of Radiology University of Cambridge Cambridge United Kingdom; ^2^ Cancer Research UK Cambridge Institute University of Cambridge Cambridge United Kingdom; ^3^ GE Healthcare Milwaukee Wisconsin USA; ^4^ MMIV, Department of Radiology Haukeland University Hospital Bergen Norway; ^5^ GE Healthcare Munich Germany

**Keywords:** carbon‐13, eddy currents, image artifacts, magnetic resonance spectroscopy, MRI, phosphorus‐31, sodium‐23, X‐nuclei

## Abstract

**Purpose:**

The aim of the study was to investigate whether incorrectly compensated eddy currents are the source of persistent X‐nuclear spectroscopy and imaging artifacts, as well as methods to correct this.

**Methods:**

Pulse‐acquire spectra were collected for ^1^H and X‐nuclei (^23^Na or ^31^P) using the minimum TR permitted on a 3T clinical MRI system. Data were collected in 3 orientations (axial, sagittal, and coronal) with the spoiler gradient at the end of the TR applied along the slice direction for each. Modifications to system calibration files to tailor eddy current compensation for each X‐nucleus were developed and applied, and data were compared with and without these corrections for: slice‐selective MRS (for ^23^Na and ^31^P), 2D spiral trajectories (for ^13^C), and 3D cones trajectories (for ^23^Na).

**Results:**

Line‐shape distortions characteristic of eddy currents were demonstrated for X‐nuclei, which were not seen for ^1^H. The severity of these correlated with the amplitude of the eddy current frequency compensation term applied by the system along the axis of the applied spoiler gradient. A proposed correction to eddy current compensation, taking account of the gyromagnetic ratio, was shown to dramatically reduce these distortions. The same correction was also shown to improve data quality of non‐Cartesian imaging (2D spiral and 3D cones trajectories).

**Conclusion:**

A simple adaptation of the default compensation for eddy currents was shown to eliminate a range of artifacts detected on X‐nuclear spectroscopy and imaging.

## 1 INTRODUCTION

Eddy currents arise when time‐varying gradients induce currents in nearby conductive structures, such as coils and the cryostat, which oppose the change in gradient field, and these currents are frequently responsible for spectroscopic and imaging artifacts. These spatially varying eddy currents accumulate because of time‐varying gradients, which can be characterized by multiple components of exponential time‐constant terms. One component of an eddy current at observation time *t* can be expressed as:

(1) $$e\left(t\right)=-\int_{0}^{t}\alpha \cdot dg/dt^{\prime }\cdot \;\exp\left(‐\beta \left(t‐t^{\prime }\right)\right)dt^{\prime },$$

where *dg/dt*′ is the rate of change of a gradient at time *t*′, α is an amplitude term, 1/β is the time constant of the eddy current, and (*t*–*t*′) is the delay between eddy current generation and observation. When the gradients are at 0 or constant amplitude, the eddy currents consist of multiple components that each decay exponentially with different time constants:

(2) $$e\left(t\right)=e\left(t_{R}\right)\cdot \exp\left(‐\beta \left(t‐t_{R}\right)\right),$$

where *t_R_* is the end of the gradient ramp.

These eddy currents generate magnetic fields, which can most easily be observed by recording an FID after the gradient has been switched off. The eddy currents cause a phase accumulation (Δϕ) between adjacent points, from which the eddy current field B can be calculated:

(3) Δϕ/Δt=ω=γB.

The unwanted effects of eddy currents on MR experiments can be counteracted by system modification to prevent or counteract their production, by post‐processing to remove the effects, or by a combination of these methods.

A highly effective post‐processing correction for eddy currents is available for ^1^H‐MRS using a separately acquired reference spectrum from the unsuppressed water to deconvolve the phase of the signal.^1^ This technique is less applicable in X‐nuclear spectroscopy, where there is no equivalent of the unsuppressed water peak as a basis for correction. Collection of separate data solely for eddy current correction would be possible^2^ but would either add to the scan time significantly for low‐SNR X‐nuclei, or would involve extrapolating corrections for X‐nuclei from ^1^H data, which may be inexact. For imaging experiments, the actual k‐space trajectories played out can be measured and compensated,^3^ but that must be repeated for each different type of imaging experiment. Finally, collection of dynamic calibration data for post‐processing correction using field cameras can be used,^4^ but this requires additional hardware.

Most modern MR scanners include extensive methods to reduce the effects of eddy currents during acquisition. On some scanners, principally preclinical devices, this involves the use of dynamic B_n_ coils, which produce an eddy current compensation that is insensitive to the gyromagnetic ratio.^5^ On others, such as General Electric (GE) devices, the gradient and frequency terms are compensated separately as follows. A calibration procedure with a complex arrangement of small phantom samples and detector coils is carried out (a field camera as mentioned above), where eddy currents are measured directly by acquiring data at a range of delays during and after application of a gradient.^6^ The measurements are made with gradients applied along the 3 orthogonal axes (*X*, *Y*, and *Z*), with multiple coils co‐located with water samples at locations offset from the isocenter along these directions, and the eddy currents are decomposed into spatially dependent and independent effects. For example, if 6 coils are used to acquire data *B_n_*(*t*) where *n* = 1:6, these components can be determined by a least squares fit to the data:

(4) $$\left(\begin{array}{c} B_{1}\left(t\right)\\ B_{2}\left(t\right)\\ B_{3}\left(t\right)\\ B_{4}\left(t\right)\\ B_{5}\left(t\right)\\ B_{6}\left(t\right) \end{array}\right)=B_{0}\left(t\right)+G_{x}\left(t\right)\cdot \left(\begin{array}{c} X_{1}\\ X_{2}\\ X_{3}\\ X_{4}\\ X_{5}\\ X_{6} \end{array}\right)+G_{y}\left(t\right)\cdot \left(\begin{array}{c} Y_{1}\\ Y_{2}\\ Y_{3}\\ Y_{4}\\ Y_{5}\\ Y_{6} \end{array}\right)+G_{z}\left(t\right)\cdot \left(\begin{array}{c} Z_{1}\\ Z_{2}\\ Z_{3}\\ Z_{4}\\ Z_{5}\\ Z_{6} \end{array}\right).$$

Following this decomposition, the B_0_ and linear effects are directly fitted with multiple exponential functions, each with a time constant and amplitude term. The spatially dependent effects caused by the eddy currents are compensated with an applied equal and opposite pre‐emphasis to the gradient waveforms along *X*, *Y*, and *Z*. Similarly, the spatially independent but time‐varying field Δ*B*_0_ (*t*) is compensated by modulating the center frequency:

(5) $$\Delta f_{0}\left(t\right)=\frac{\gamma }{2\pi }\Delta B_{0}\left(t\right).$$

The experiments are repeated with these corrections applied, new fits are performed to the residual eddy currents measured, the correction terms are modified, and this iterative cycle is repeated until the residual eddy currents are below an acceptable threshold.

Although the eddy currents themselves are independent of field strength, precession frequency is proportional to the gyromagnetic ratio, therefore, it is important that any frequency correction should take this into account. In our experience, visual inspection of data has often suggested a possible contribution from eddy currents to the poor quality of X‐nuclear spectra and images. Spectral peaks have been distorted, and images have been distorted or displaced relative to anatomic ^1^H scout images. However, subsequent ^1^H‐MRS failed to demonstrate a problem, despite its sensitivity to eddy current effects in general.^1^ It is, therefore, likely that eddy current effects on X‐nuclei may be overlooked on human imaging systems where the focus is optimizing ^1^H‐MRI quality for routine clinical use and that some modifications for different gyromagnetic ratio may be needed for optimal acquisition.

In this paper, we present a method to characterize the severity of eddy current artifacts on different nuclei, and we demonstrate a fix on a clinical system that greatly improves the quality of X‐nuclear images and spectra.

## 2 METHODS

Experiments were performed on a 3T MR750 MR system (software version DV26.0_R02, GE Healthcare, Waukesha, WI). Acquisition was performed with quadrature birdcage head coils (Rapid Biomedical, Rimpar, Germany) dually resonant for ^1^H (128 MHz) and either ^23^Na (33.9 MHz), ^13^C (32.1 MHz), or ^31^P (51.7 MHz).

A 16‐cm diameter MRS head sphere phantom (GE Healthcare) was used for ^1^H, ^23^Na, and ^31^P spectroscopy. For ^13^C, a 3‐cm diameter sphere of 1 M ^13^C‐labeled bicarbonate was used (GE Healthcare), because natural abundance phantoms would have given low signal, often complicated by J‐coupling with nearby ^1^H nuclei. To assess the performance of sodium 3D cones acquisition, a gradient calibration phantom (DQA III, GE Healthcare) was emptied and refilled with 80 mM sodium chloride solution.

Slice‐selective pulse‐acquire spectra with matched parameters were collected for ^1^H and X‐nuclei (^23^Na, ^31^P, and/or ^13^C) using the minimum allowed TR (420 ms) and with incrementally increased delays between the large spoiler gradient at the end of 1 TR period and the beginning of the next. Data were collected in 3 orientations (axial, sagittal, and coronal) with the spoiler gradient at the end of the TR applied along the slice direction. The spoiler gradient amplitude = 22 mT/m, pulse width = 6.5 ms, ramp = 0.42 ms, and area = 0.152 mT s m^−1^.

As described in the Introduction, eddy current correction on this system involves applying pre‐emphasis to the frequency and the gradient waveforms using multiple exponential terms, with a time constant and amplitude for each. The amplitude terms (α) of the frequency modulation for eddy current correction were altered in the following ways by creation of modified system calibration files: they were set to 0, or they were rescaled for the gyromagnetic ratio of each X‐nucleus according to:

(6) $$\alpha_{X}\;=\;\alpha_{\text{1H}}\;*\;\gamma_{X}/\gamma_{\text{1H}}.$$

Swapping between these system calibration files was performed within an examination by the execution of a Python script. Data were collected and compared with these different calibration files using slice‐selective MRS, 2D spirals^7^ (single‐shot 60‐point spiral, FOV = 24 cm, nominal pixel size = 4 mm, 2‐cm thick slice, 90° flip angle, 128 averages, TR = 75 ms, 0:10 min), and 3D cones^8^, ^9^ (24 cm FOV, nominal pixel size 3 mm isotropic, TR = 45.6 ms, TE = 0.46 ms, hard pulse flip angle 90°, 1 average, 2552 interleaves, 1:56 min).

Spectra and images were reconstructed and displayed in MATLAB (The MathWorks, Natick, MA) and SAGE (GE Healthcare). They were assessed qualitatively for the presence and severity of eddy current artifacts, and the size of the eddy current induced frequency shift was measured as the displacement in Hz between the peak in the last transient in a series of 8 spectra, which was distorted by the spoiler gradients in the previous repetitions, and the first spectrum, which was not preceded by a spoiler gradient and was, therefore, unaffected.

## 3 RESULTS

**FIGURE 1:**
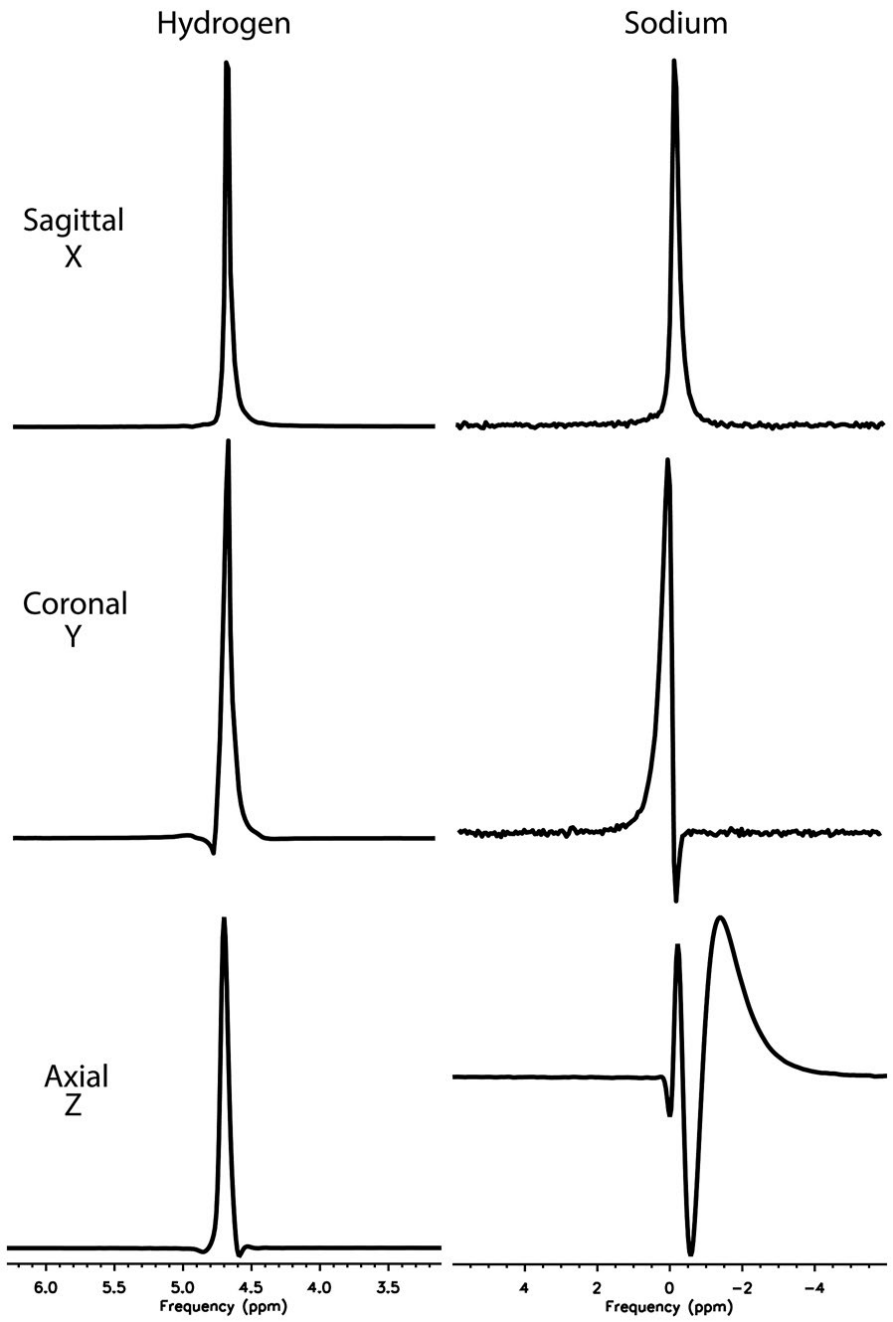
Eddy current distortions in a phantom spectrum acquired using ^1^H‐MRS and ^23^Na‐MRS. Slice‐localized MRS was acquired along each orthogonal axis in a large spherical saline phantom (5000 Hz, 2048 points) with application of the default correction for eddy currents. Although only minor distortion is seen in the axial orientations on ^1^H (left), marked distortions typical of eddy currents are seen in a ^23^Na spectrum from the same slice (right), whereas there is less distortion for ^23^Na in the coronal orientation, and virtually none in the sagittal orientation

In pulse‐acquire spectroscopy at the minimum TR, small distortions because of eddy currents were seen in ^1^H MR spectra of water acquired in axial and coronal orientations (with crusher gradients applied along Z and Y, respectively), but not when the crusher gradients were applied along X (sagittal orientation; Figure 1). When spectra were acquired from the same phantom at ^23^Na frequency, there was still no evidence of eddy currents in the sagittal orientation, but the distortion was greater than seen for ^1^H in the coronal orientation, and very much greater than ^1^H in the axial orientation.

**FIGURE 2:**
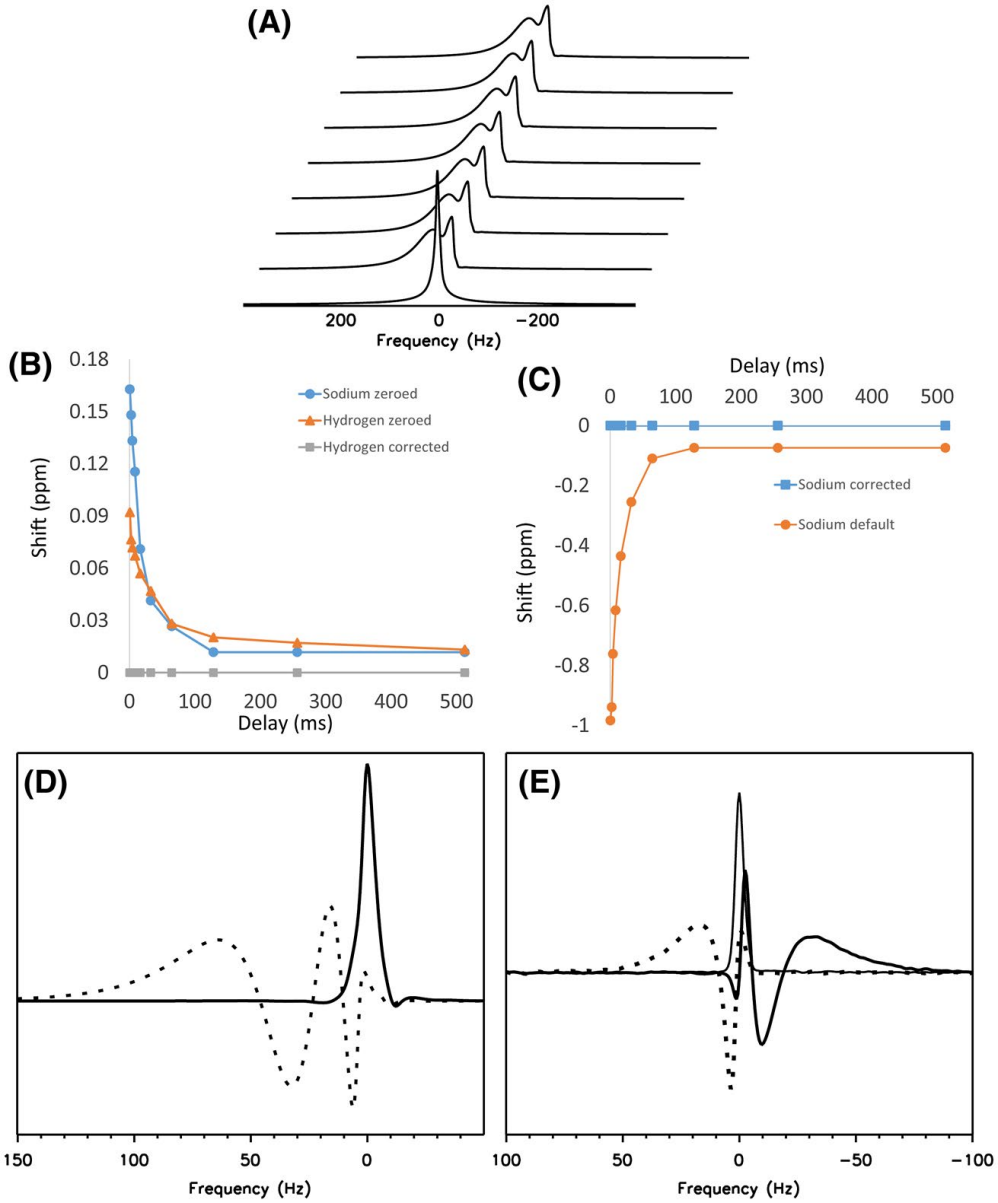
Eddy current distortions with frequency modulation disabled and modified. (A) A stack of ^1^H‐MR spectra acquired with zeroed frequency modulation, in an axial orientation at the minimum TR (420 ms). The first acquisition in the series is free of distortion, whereas subsequent acquisitions show frequency shifts and phase distortion caused by the spoiler gradients applied in the previous TR periods. (B) The frequency shift (ppm) between the tallest peak in the first and last spectra in a stack as shown in (A) was measured at a range of time delays between the end of the spoiler gradient in 1 TR period and the excitation in the next, for both hydrogen and sodium with zeroed f_0_ correction terms, and for hydrogen with the default correction applied. (C) As in (B), frequency shifts are shown for sodium with the default f_0_ terms and with the corrected terms recalculated for the gyromagnetic ratio. (D) ^1^H‐MRS acquired using a ^1^H/^31^P head coil, with either zeroed frequency correction (dotted line) or default ^1^H‐optimized correction (solid). (E) ^31^P‐MRS acquired with the same coil and phantom, using frequency corrections: zeroed (dotted line); default ^1^H‐optimized (thick line); custom ^31^P‐optimized (narrow line)

To investigate this further, we modified the system calibration files such that the gradient pre‐emphasis was still applied but the frequency modulation amplitude terms were set to 0. Pulse‐acquire spectroscopy then showed larger eddy current effects on ^1^H than on X‐nuclei, particularly in the Z direction (Figure 2).

An example of how these relative shifts varied with increasing delay between the spoiler pulse at the end of 1 TR period and the start of the next is shown in Figure 2B,C. The rate of decay with increasing time delay appeared very similar although not identical between ^1^H and ^23^Na, and the magnitude of the frequency shifts was roughly in proportion to the gyromagnetic ratios. Although the frequency shifts with zeroed correction were largest in Hz for ^1^H, when rescaled to ppm they were similar between nuclei. Applying default frequency corrections for X‐nuclei resulted in clear overcompensation, because the magnitude of frequency shift became much larger and changed sign (Figure 2C).

We further confirmed that the existing frequency modulation produced an overshoot for a different X‐nucleus (phosphorus) and that a simple scaling of the amplitude coefficients for modulation could dramatically improve the data (Figure 2D,E). In Figure 2D, the existing correction worked well for ^1^H: with zeroed frequency modulation, severe distortions were seen and the tallest peak in the magnitude spectrum was shifted by +7.0 Hz; when the default ^1^H‐optimized frequency modulation was applied, the lineshape was much improved and the shift was 0.0 Hz. For the phosphorus acquisition (Figure 2D), the zeroed modulation produced a shift in the same direction as ^1^H but smaller, +3.1 Hz; the default ^1^H‐optimized f_0_ correction produced a shift in the opposite direction, −3.1 Hz; and the f_0_ correction with amplitudes rescaled for ^31^P gave a shift of 0.0 Hz and an undistorted lineshape.

**FIGURE 3:**
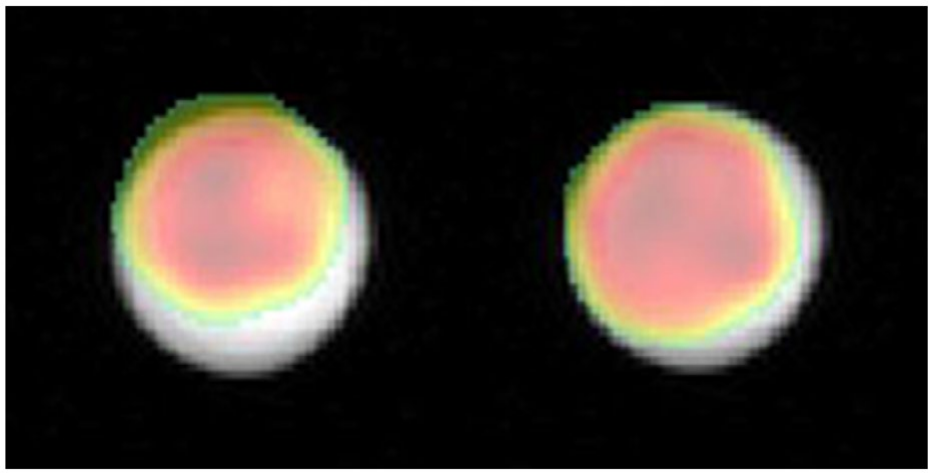
Spatial misregistration of spiral X‐nuclear images. With the default eddy current correction settings (left), a coronal spiral image of a spherical phantom of 1 M ^13^C‐bicarbonate (overlaid in color) is seen to be offset in the superior‐inferior direction relative to the corresponding ^1^H‐MRI image. With corrected eddy current f_0_ terms (right), this offset is reduced. For clarity, only the central 6 cm of a 24‐cm FOV are shown

The original impetus for exploring the eddy current behavior had been the observation that low frequency, non‐Cartesian X‐nuclear images were consistently displaced superiorly relative to an anatomic ^1^H image. This is shown in Figure 3 with coronal spiral images, using a spherical phantom of labelled ^13^C‐bicarbonate. When the rescaled f_0_ correction terms are used for compensation, the displacement is reduced.

**FIGURE 4:**
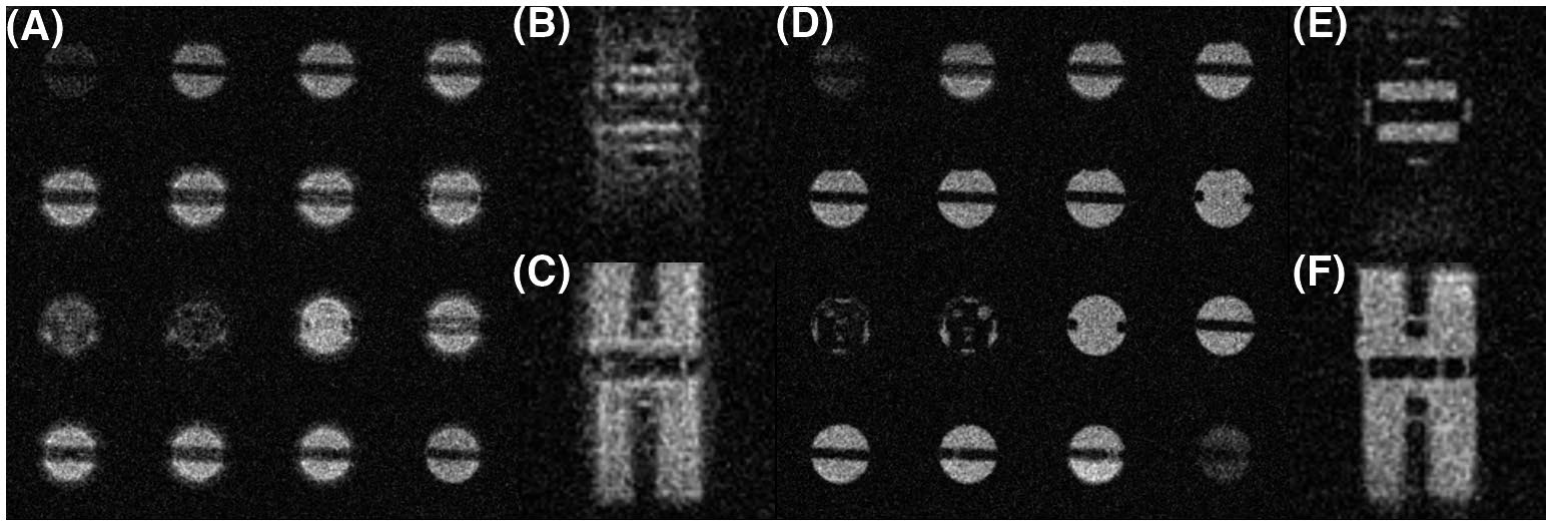
Distortion of X‐nuclear cones images. (A) With the default eddy current correction settings, a series of ^23^Na axial 3D cones images of a phantom of 80 mM saline acquired at short TR (46 ms) is seen to be blurred and distorted. This is particularly evident in the coronal (B) and sagittal (C) projections. With corrected eddy current f_0_ terms (D–F), the blurring and distortion are greatly reduced

Similar displacement effects were observed when imaging sodium using 3D non‐Cartesian sequences such as cones. Additionally, when using a short TR there was considerable blurring and distortion: rescaling the eddy current f_0_ correction terms for sodium was shown to improve image quality markedly in a 3D cones data set (Figure 4).

## 4 DISCUSSION

Spectroscopy using a spoiler gradient and the minimum allowed TR presents a simple, quick and effective method to check the relative performance of eddy current compensation for different nuclei and axes. This approach is less sensitive and precise than the methods used to calibrate the system for ^1^H: those experiments use very long gradient pulses to diminish the interference between eddy currents caused by turning the gradients off and those caused by turning them on. Common ^1^H methods also use a field camera, whereas for many X‐nuclei the SNR obtainable in a rig of tiny samples might be insufficient for full multi‐exponential fitting. The advantage of the method used in the current study is that it can be performed without specialist equipment and without changing the default pulse sequence variables, which should increase the robustness for inter‐site comparisons. As shown in Figure 1, the method still retains sufficient sensitivity to detect and characterize problems.

We demonstrated that on a GE MR750 MRI scanner, eddy current compensation was significantly worse for X‐nuclei than for ^1^H, which is likely to be because of the applied frequency modulation for eddy currents. Because this modulation was the same for all nuclei regardless of their resonant frequency, it is expected to over‐correct the lower‐frequency X‐nuclei. We have confirmed that zeroing this frequency modulation produced lineshape distortions and frequency shifts for ^1^H that were greater than for the X‐nuclei (Figure 2). Application of the default ^1^H‐optimized f_0_ correction compensated the ^1^H acquisition adequately, but produced an overshoot in the frequency shift of ^31^P and ^23^Na. Correction of this error was demonstrated using a simple rescaling of a series of amplitude coefficients in an eddy current compensation file by the ratio of gyromagnetic constants for the X‐nucleus relative to hydrogen. A separate correction must be used for each nucleus because the degree of over‐compensation is dependent on the gyromagnetic ratio. We predict that this effect would be minor for ^19^F because of its similar frequency to ^1^H, and that artifacts would worsen progressively for ^31^P, ^23^Na, ^13^C, and ^2^H. It should be noted that this error is specific to the method of f_0_ eddy current compensation used; it would not be expected to occur where the correction was applied through the use of zeroth order field coils, which we believe to be more common on pre‐clinical systems.

Although it appeared that the time constants as well as amplitude coefficients of the eddy current terms might vary slightly between nuclei (Figure 2B), these apparent differences were probably caused by the imprecision of quantifying small frequency shifts by fitting a single frequency to broad peaks using limited spectral resolution. This limitation prevents confident multi‐exponential fitting of the curves produced, which, could in theory, be used to re‐derive the f_0_ correction parameters for each X‐nucleus individually. Additionally, the artifacts we observed were empirically well corrected by adjusting amplitude coefficients only (Figures 2C and 2E); therefore, the time constants were assumed to be the same for all nuclei.

Application of these corrections was shown to improve the quality not only for spectroscopy, but also for fast imaging of X‐nuclei using spiral and 3D cones sequences. These techniques are widely used in clinical research, particularly for ^23^Na,^10^ hyperpolarized ^13^C,^11^ and ^129^Xe.^12^ Overcompensation of eddy current f_0_ terms may have had quite widespread effects on signal localization and quantification using these approaches. Other fast imaging sequences such as EPI would also be expected to suffer from overcompensated eddy currents, but they are not implemented for X‐nuclei on our system and were not tested. Diffusion‐weighted (DW) MRS of X‐nuclei on clinical systems^13^ would be expected to be especially sensitive to eddy currents: a pulse sequence to perform DW‐MRS was implemented in 2014 on our system but abandoned because of the problems experienced in particular when applying gradients along the Z axis for X‐nuclei. This is consistent with the effects seen in Figure 1 and the relative eddy current correction coefficients on our system.

The results reported here are limited to a single MR scanner from a single manufacturer (GE), and it seems that not all GE scanners are equally affected. Lu et al^3^ concluded that because ^1^H and ^23^Na were affected approximately equally by eddy currents on their GE 3T scanner, they could correct ^23^Na based on ^1^H calibrations. However, there are indications that some scanners from other manufacturers may also be affected.^14^

It should be noted that the experiments that show this artifact most clearly are not often performed as part of human studies. MR spectroscopy is rarely acquired at the minimum possible TR, because T_1_s of metabolites of interest tend to be long, such that SNR per unit time is maximized by acquiring at longer TRs. Inversion‐recovery MRS experiments with short inversion times are more commonly undertaken, given their usefulness in estimating T_1_, but few clinical studies of quantitative T_1_ relaxometry have been reported on X‐nuclei. Additionally, the error is most obvious when the uncorrected Δ*f*_0_(*t*) term is large to begin with (Figure 1). Where the frequency shifts because of eddy currents are small on ^1^H, even a fourfold overcompensation (as is the case for ^13^C, where the gyromagnetic ratio is 4 times smaller) may produce only subtle effects.

A temporary solution of tailoring calibration files for each nucleus was demonstrated to completely eliminate evidence of eddy current artifacts on a clinical system; however, this carries a risk to the clinical service if operators fail to reset these for subsequent ^1^H acquisition. Automation of this fix within the system applications software would be desirable, to eliminate the need for user intervention. We have demonstrated that such a fix has the potential to markedly improve multi‐nuclear image quality.

## CONFLICT OF INTEREST

Rolf Schulte is an employee of General Electric. Scott Hinks is a retired employee of General Electric.
